# Effect of Instant Controlled Pressure-Drop on the Non-Nutritional Compounds of Seeds and Sprouts of Common Black Bean (*Phaseolus vulgaris* L.)

**DOI:** 10.3390/molecules25061464

**Published:** 2020-03-24

**Authors:** Anaberta Cardador-Martínez, Yara Martínez-Tequitlalpan, Tzayhri Gallardo-Velazquez, Xariss M. Sánchez-Chino, Jorge Martínez-Herrera, Luis Jorge Corzo-Ríos, Cristian Jiménez-Martínez

**Affiliations:** 1 Departamento de Bioingenierías, Tecnologico de Monterrey, Av. Epigmenio González No. 500, Fraccionamiento San Pablo, Querétaro 76130, Mexico; 2 Escuela Nacional de Ciencias Biológicas, Instituto Politécnico Nacional, Av. Wilfrido Massieu Esq. Cda. Miguel Stampa s/n, Delegación Gustavo A. Madero, México City, CdMx 07738, Mexico; yaribq@hotmail.com (Y.M.-T.); gtzayhri@yahoo.com (T.G.-V.); 3 Cátedra-CONACyT, Departamento de Salud, El Colegio de la Frontera Sur-Villahermosa, Carretera a Reforma Km. 15.5 s/n. Ra. Guineo 2da. Sección, Villahermosa, Tabasco 86280, Mexico; xsanchez@mail.ecosur.mx; 4 Instituto Nacional de Investigaciones Forestales, Agrícolas y Pecuarias, Tabasco, Campo Experimental Huimanguillo, Km. 1. Carr. Huimanguillo-Cárdenas, Tabasco 86400, Mexico; martinez.jorge@inifap.gob.mx; 5 Unidad Profesional Interdisciplinaria de Biotecnología, Instituto Politécnico Nacional, Av. Acueducto S/N, Barrio La Laguna, Col. La Laguna Ticomán, México City 07340, Mexico; lcorzor@hotmail.com

**Keywords:** germination, controlled pressure drop (DIC), black beans, non-nutritional compounds, phenolics, phytates, oligosaccharides, trypsin inhibitors

## Abstract

The common bean is an important caloric-protein food source. However, its nutritional value may be affected by the presence of non-nutritional compounds, which decrease the assimilation of some nutrients; however, at low concentrations, they show a beneficial effect. Germination and treatment by controlled pressure-drop (DIC, French acronym of *Détente Instantanée Contrôlée*) are methods that modify the concentration of these components. The objective of this work was to evaluate the change in the non-nutritional composition of bean seeds and sprouts by DIC treatment. The results show that with the germination, the concentration of phenolic and tannin compounds increased 99% and 73%, respectively, as well as the quantity of saponins (65.7%), while phytates and trypsin inhibitors decreased 26% and 42%, respectively. When applying the DIC treatment, the content of phytates (23–29%), saponins (44%) and oligosaccharides increased in bean sprouts and decreased phenolic compounds (4–14%), tannins (23% to 72%), and trypsin inhibitors (95.5%), according to the pressure and time conditions applied. This technology opens the way to new perspectives, especially to more effective use of legumes as a source of vegetable protein or bioactive compounds.

## 1. Introduction

The common bean (*Phaseolus vulgaris* L.) is one of the most important legumes in human food due to its high nutritional value. However, its consumption can be limited by the high concentration of some secondary metabolites, naturally synthesized in the plant and which, due to the adverse effect they may present, have been referred to as non-nutritional compounds [1,2]. Among these are phenolic compounds, tannins, phytic acid, protease inhibitors, saponins, and oligosaccharides [3]. These compounds are synthesized and accumulated during the maturation of the seed for the germination process, or as a defense mechanism (protease inhibitors, lectins, tannins, L-DOPA) against the attack of bacteria, viruses, fungi, insects and animals, including man [4], and when ingested as part of food can reduce the availability of some nutrients of interest, such as carbohydrates, proteins, vitamins, and minerals, causing an undesirable physiological side effect (flatulence) and generating neurotoxic effects when consumed in high amounts [5,6].

Another benefit of legume seeds is that they can be dried and stored for long periods, so they are important for food safety. For their consumption they must go through different processes to rehydrate and soften the cotyledons, in this way their consumption is facilitated, their nutritional profile and organoleptic properties are improved, besides eliminating, reducing, or inactivating non-nutritional factors [2,5,7]. Among the processes are soaking, cooking, fermentation, germination, and combinations of these [8,9].

The germination process begins with the imbibition and ends with the emergence of the radicle. During this process, there is a set of metabolic and morphological changes, activation of transcription and translation in the seed [10]. Sprouts are nutritionally superior to their original seeds with higher levels of nutrients, lower amounts of antinutrients, and increased protein and starch digestibility [11]. All these changes are influenced by external factors such as the kind of legume, germination conditions, presence or absence of light and germination time. Although during germination there is a reduction of non-nutritional compounds, it is no significant, then combinations of germination with other treatments have been evaluated.

The treatment by controlled pressure-drop (DIC, French acronym of *Détente Instantanée Contrôlée*) is a technology used to accelerate the drying process. DIC is a thermo-mechanic treatment at high temperature and short time (HTST), combined with a sudden pressure drop. The material processed by DIC has a porous structure and crunchy taste among other organoleptic characteristics [12,13]. DIC technology has been used to reduce and eliminate microorganisms, preserving organoleptic properties of fruits, vegetables, cereals, and meat to guarantee the quality of these products in function of the operation conditions (temperature, pressure, and time) [13,14].

Haddad and Allaf [9] studied the effect of DIC over soy trypsin inhibitors concentration, and observed a 94% reduction when the processing conditions were humidity of 20–57%, treatment 30–60 s and pressure 1.57–7.89 atm. 

Pedrosa, et al. [6] evaluated the effect of DIC (pressure of 2.96 and 5.92 atm, and time of 1 and 3 min) on the content of oligosaccharides, inositol phosphate, trypsin inhibitors and hemagglutinins of lupins soy, pea, lentils, and peanuts. These authors reported an increment in the content of oligosaccharides, while the concentration of the other non-nutritional compounds evaluated was reduced.

Cuadrado, et al. [15] applied DIC to reduce the allergenicity of peanuts, lentils, chickpea. and soybean, using 3 and 6 bar of pressure during 1 and 3 min. Although the total content protein was no modified, there was a general decrease in IgE binding to legume proteins that was correlated to higher steam pressure and more prolonged treatment.

This study aimed to evaluate the effect of DIC on the concentration of non-nutritional compounds of black bean sprouts (*Phaseolus vulgaris* L.).

## 2. Results and Discussion

In the next sections, the content of non-nutritional compounds is described in black beans without treatment (BNT), black bean sprouts after 7 days of germination and lyophilized (BGL) and also, germinated seeds dried at 50 °C (BGD).

### 2.1. Non-Nutritional Compounds in Black Bean without Treatment (BNT), Germinated-Lyophilized (BGL), and Germinated-Dried (BGD)

The content of total phenolics BNT was 4.11 mg Gallic acid equivalent per g of dry sample (Table 1). This content was in the range of previous results (4.15–13.26 mg/g dry basis). The slight differences could be attributed to genotype, water content, soil acidity or alkalinity, temperature among others [16,17].

Pająk, et al. [18] reported that after germination total phenolic content increased by 99% (until 8.2 mg/g dry basis. Other authors have reported that there is an increase in hesperetin, 7,3′,4′-trihydroxyflavone, 8-hydroxydihydrodaidzein, and 6-hydroxydaidzein during the germination of chickpea and lentil [19]. Moreover, Fernandez-Orozco, et al. [20] reported that sprouts of mung bean and soybeans provided more total phenolic compounds than did raw seeds. High increments in phenolic compounds of dark mungo bean were obtained when germination was elicited by fish protein hydrolysates, lactoferrin, and oregano extract suggesting that elicitors were responsible for the improvement in phenolics content [21]. On the other hand, a reduction in free phenolic compounds (afzelequin, prunetin, formononetin, and glicitein) has been observed probably due to their consumption during the germination of the seed due to its normal physiological activity [19,22].

None of the non-nutritional compounds’ contents presented differences between BGD and BGL (*p* < 0.05), suggesting that the drying method had no effect over these compounds.

The content of phytic acid in BNT was 17.33 mg eq/g db (Table 1). Oloyo (2004) reported a range of 7–13 mg/g in pea, lentils, and chickpea without treatment. After germination, phytic acid concentration decreased until 12.8 mg eq/g, equivalent to a 26.5 reduction. Sangronis and Machado (2007) reported an initial content of 7.8, and 9.11 mg/g of phytic acid in navy and black beans, and observed reductions of 45 and 52% after five days of germination. In lentils, the decrease in phytic acid concentration depends on germination time [23]. Fouad and Rehab [24] reported an increase in the reduction of phytic acid content from day 3 to day 6 (45% and up to 74%, respectively). The decrease in phytic acid content could be due to the activity of phytase, this enzyme is expressed during germination to provide the necessary compounds for the development and survival of the new plant. Phytase produces compounds with less content of phosphate, making phosphate more bioavailable [24,25,26,27].

Condensed tannin concentration was 2.62 mg/g db in *P. vulgaris* L. (Table 1). The germination process increased by 73% condensed tannins, independently if germinated beans were lyophilized or oven-dried. Rusydi, et al. [28] reported a 28% and 64% reduction in the tannin content of soy and peanut, respectively, after germination. 

Saponin content increased from 7.05 to 9.37 mg diosgenin eq/g of the sample due to the germination process. The drying method did not modify saponin content (Table 1). Paucar-Menacho, et al. [29] reported similar initial content of saponins in soybean (7.4 mg/g), and depending on the germination time; saponins varied from 6.7 to 23.5 mg/g. The increase in saponin content during germination could be due to the synthesis and activation of different enzymatic systems responsible for the production of secondary metabolites, the weakening, and the modification of the seed structure.

On the other hand, trypsin inhibitors decrease when black beans were germinated, by approximately 46%. Trugo, et al. [30] reported a value of 98 TIA/g db in black beans, while Pedrosa, et al. [6] found 86.1 TIA/ g db in soybean; these values were like the one in this study. Sangronis and Machado [31] reported a decrease in trypsin inhibitor of 52.5 and 25.6% in germinated navy and black beans, respectively. Germination is a catabolic process that uses reserve compounds contained in cotyledons to sustain the growth of the plant; trypsin inhibitors could be used as an energy source. Higher temperatures such as autoclaving, boiling or microwave produced reductions in trypsin inhibitors of 83.87%, 82.27%, and 80.5% in chickpea [32]. Moreover, Sánchez-Chino, et al. [33] reported a 97% reduction in trypsin inhibitors after soaking and cooking of chickpea.

Carbohydrates account for 55% to 65% of the legume grain (Hangen and Benkini, 2002), being oligosaccharides the more abundant after starch. In this study, the most abundant oligosaccharide was stachyose, with content around five times the one of raffinose and verbascose (Table 1) in BTN. After germination, raffinose content was increased up to 28.95 mg/g, while stachyose decreased by 41% and verbascose was not detected.

### 2.2. Effect of DIC on the Non-Nutritional Compounds of Black Bean Sprouts

Among secondary metabolites, phenolics are remarkable because of their biological activity, for instance, antioxidant, that is why that independently of the process, phenolics should not change their concentration.

**Figure 1:**
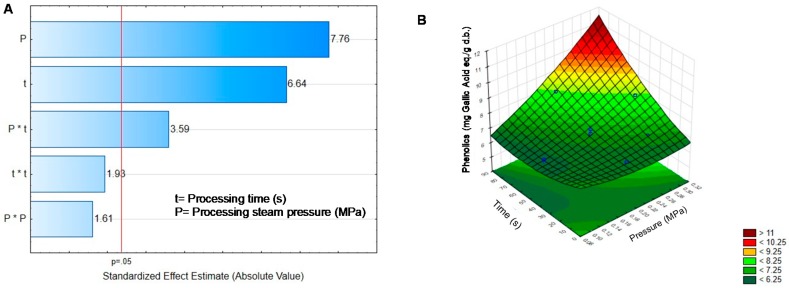
Effect of temperature and pressure over the content of Phenolics (**A**): Pareto Chart. (**B**): Response surface. Critical values to obtain the highest phenolic concentration predicted by the model P = 0.17 MPa, t = 13.79 s. Phenolics = 8.03 − 12.71P + 26.39t^2^ − 0.05t + 0.00025t^2^ + 0.265P × t.

Table 2 shows the content of non-nutritional compounds in black sprouts dried at 50 °C and treated by DIC. Phenolics did not show a tendency, although DIC treated samples are significantly different from BGD; some DIC treatments increase the phenolic content slightly, while others did the contrary. DIC treatment involves the application of steam to sprouts; the temperature in the processing chamber could reach 100–120 °C. Changes in phenolics during DIC treatment were related to both, temperature and pressure, and their quadratic effect, as well as the interaction of these factors as shown in Figure 1A.

Alonzo-Macías, et al. [34] and Gahler, et al. [35] reported that DIC treatment modifies the structure of biological material, causing the opening of cellular structures, making phenolics more available, and then an increase in phenolics concentration could be observed. Moreover, Stewart, et al. [36] suggested that thermal treatment produces the rupture of cell walls, causing the release of phenolic compounds. Not only high temperatures but also low ones can increase phenolic content after germination as demonstrated by Świeca and Gawlik-Dziki [37] who observed an increase in phenolics of green pea, lentil, and mung bean sprouted and storage under cool condition. The high concentration of phenolics was obtained at a short time and low pressure, as can be seen in Figure 1B. According to Mubarak [38], high temperatures applied on legumes induce the loss of soluble solids and reduce or eliminate non-nutritional factors such as phenolics.

**Figure 2:**
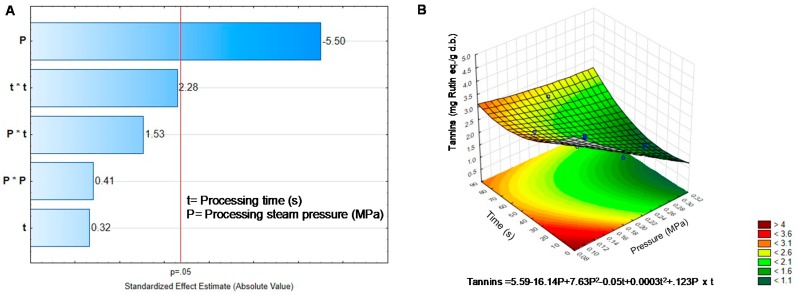
Effect of temperature and pressure over the content of Tannins (**A**) Pareto Chart. (**B**) Response surface. Critical values predicted by the model P = −0.91 MPa, t = 242.77 s.

On the other hand, Tannins decrease their concentration significantly after DIC treatment (Table 2). The final concentration in most DIC treated sprouts was around half the initial content in BGD. The highest reduction in tannins was achieved when high pressure (0.3 MPa) was applied and medium to long times (45–70 s). Reduction in tannins was modified by pressure, while temperature, the interaction, and the quadratic factors were no significant, as seen in Figure 2A.

Respect condensed tannins, a reduction from 23% up to 72% was observed depending on the operation condition (Table 2). The higher the pressure, the higher the reduction; therefore, pressure (Figure 2A), was responsible for the decrease in tannins. However, the surface response and the equation could not predict the critical values to get a high reduction in tannins (Figure 2B). It should be necessary to look for a linear adjust to predict the pressure value to reach an efficient reduction of tannins. According to Shimelis and Rakshit [27] the reduction in tannin concentrations could be due to solubilization in condensed vapor and posterior lixiviation during DIC treatment. Loos in tannin content could also be related to temperature reached in the DIC reactor, around 120 °C. Khandelwal, et al. [39] observed a reduction in tannins content (25–59%) in beans and lentils cooked under pressure (0.1 MPa, 121 °C).

On the other hand, Sánchez [40] evaluated the tannin content in whole, cotyledon and hull cooked beans, suggesting that tannins were reduced by 100% after thermal treatment, and the reduction in whole seed and shell was of 84.66% and 86.23% respectively.

**Figure 3:**
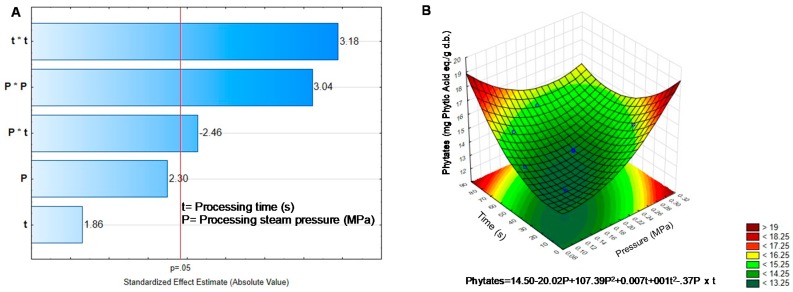
Effect of temperature and pressure over the content of Phytates. (**A**) Pareto Chart. (**B**) Response surface. Critical values predicted by the model P = 0.13 MPa, t = 24.06 s.

Phytates tend to increase their concentration as well as saponins (Table 2). An average increase of 15% was observed in phytates after DIC treatments (*p* < 0.05). A combination of low pressure (0.13 MPa) and average time (24 s, Figure 3B) caused the highest increase in phytates concentration. The quadratic effect of pressure and time as well as their interaction modified phytates content (Figure 3A), which could explain the increment in phytates content. Haddad, et al. [8] mentioned that phytates could partially be eliminated through thermal treatment. Furthermore, Pedrosa, et al. [6] found a 41% reduction in lupins phytates, 37% in lentils, 27% in chickpea, and 10% in peanut after applying 0.3 MPa during 3 min. While Haddad, et al. [8] reported a decrease of 16% and 19% phytate concentration in *Lupinus albus* and *Lupinus mutabilis* that were DIC-treated by 0.7 MPa for 7 min. In this study, germination decreases the phytates content, but DIC caused a slight increase dependent on pressure and time, suggesting that the DIC process made phytates more available. However, the final phytate concentration was lower than the initial one in black bean (Table 1).

**Figure 4:**
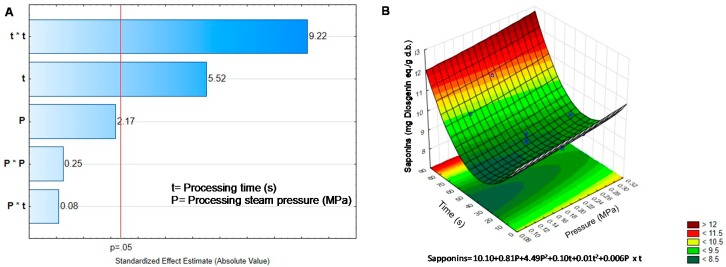
Effect of temperature and pressure over the content of Saponins. (**A**) Pareto Chart. (**B**) Response surface. Critical values predicted by the model P = 0.10 MPa, t = 38.06 s.

Saponins increased approximately 25% compared to BGD (Table 2). However, not all the treatments increase saponin content; the center points of the DIC design did not change saponin content (0.2 MPa, 45 s). In this case, only temperature and its quadratic interaction showed effect on saponin concentration according to the Pareto chart (Figure 4A). The response surface and its corresponding equation predicted that the critical values to reach the highest concentration of saponins were low pressure (0.1 MPa) and average time (38 s) (Figure 4B).

Guajardo-Flores, et al. [41] reported that saponins are present in low quantity in common beans. These saponins could be classified into three groups—A, B, and E—related to aglycone structure. B aglycones are thermolabile and can be easily degraded due to a weak covalent bond. Probably, saponins degraded after DIC treatment could be type B. 

Alonzo-Macías, et al. [34] mentioned that during DIC treatment, the pressure drop could induce expansion of the food matrix, leaving a porous structure that could increase the availability of compounds, improving food quality and preserving its nutraceutical value. Reim and Rohn [42] reported an increase of 10% in saponin concentration in peas after thermal treatment (60 °C, 4 h).

**Figure 5:**
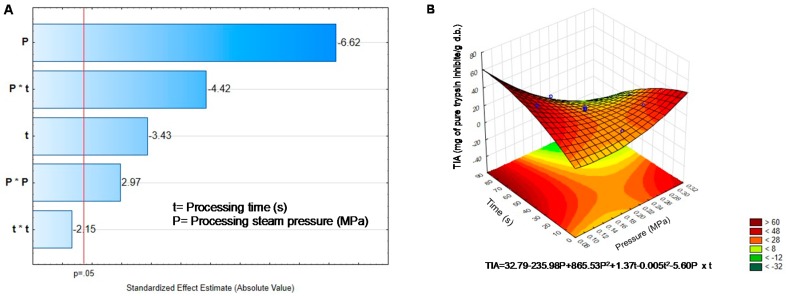
Effect of temperature and pressure over the content of Trypsin inhibitors. (**A**) Pareto Chart. (**B**) Response surface. Critical values predicted by the model P = 0.20 MPa, t = 21.42 s.

Table 2 shows a significant decrease (*p* < 0.05) in TIA concentration in all DIC treatments. Furthermore, only the quadratic interaction of time had no effect in reducing TIA content. The pressure was the factor with the most significant impact on TIA content (Figure 5A). The response surface graph and the equation model predicted that a pressure of 0.2 MPa and 21.42 s were enough to reach a high concentration of TIA (Figure 5B). The decrease in TIA content confirmed their sensitivity to heat [9].

On the other hand, to achieve the highest reduction (95.5%) in TIA content it was necessary to apply 0.27 MPa during 70 s. Similar results were obtained by Haddad and Allaf [9] in soybean treated during 60 s at 0.7 MPa. Besides Pedrosa, et al. [6] reported reductions of 94%, 96.7%, and 43.75% for soy, lentils, and chickpea, respectively. However, Avilés-Gaxiola, et al. [43] stated that a reduction between 70% and 87% in TIA content is satisfactory and Haddad and Allaf [9] that a TIA content f 1.5–3 IU/mg db is safe for human consumption.

### 2.3. Determination of α-Galactosides in Black Bean Sprouts Treated with DIC

**Figure 6:**
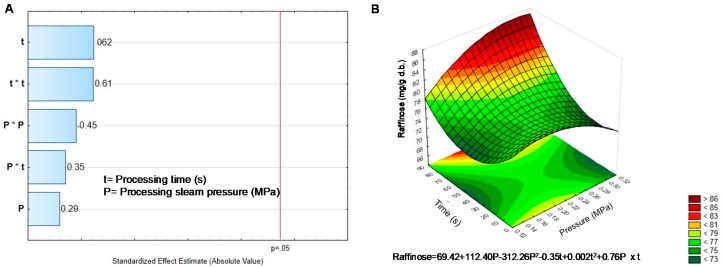
Effect of temperature and pressure over the content of Raffinose. (**A**) Pareto Chart. (**B**) Response surface. Critical values predicted by the model P = 0.22 MPa, t = 32.21 s.

**Figure 7:**
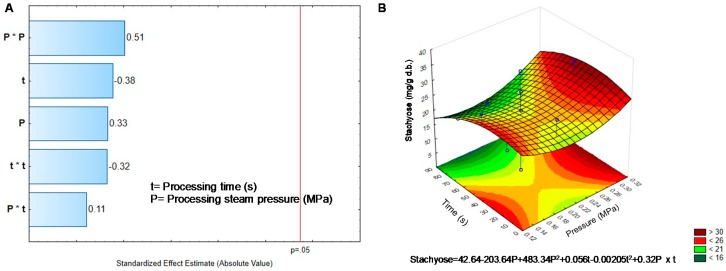
Effect of temperature and pressure over the content of Stachyose. (**A**) Pareto Chart. (**B**) Response surface. Critical values predicted by the model P = 0.20 MPa, t = 30 s.

The content of α-galactosides after DIC treatment of black bean sprouts is shown in Table 3. Both, raffinose and stachyose increase their content, possibly due to the modification of the seed structure, which after DIC treatment is more porous, and cell walls could be broken, leading to a major contact between the seed and the extraction solvent [6]. Raffinose increase its content by double, while stachyose content increases up to 80%. As shown in Figure 6A and Figure 7A, neither pressure nor time and their interaction modified the oligosaccharides content.

The models generated by the surface response graph for raffinose, predicted that critical values to obtain the higher concentration was 0.22 MPa and 32 s Figure 6B. The behavior of stachyose was very similar; even the critical values were almost the same (Figure 7B).

Amor, et al. [44] studied the effect of DIC over the extractability of ciceritol an stachyose from seeds of *Tephrosia purpurea* finding a positive effect of pressure, meaning that high pressure lead to major extraction.

Shimelis and Rakshit [27] reported that the oligosaccharides content of kidney bean was modified by processing methods such as cooking, autoclaving, and their combination due to the oligosaccharide stability at high temperatures. The increase in availability in black bean sprouts could make them a good source of oligosaccharides, which could be used as valuable ingredients or probiotics [45].

### 2.4. Principal Component Analysis

**Figure 8:**
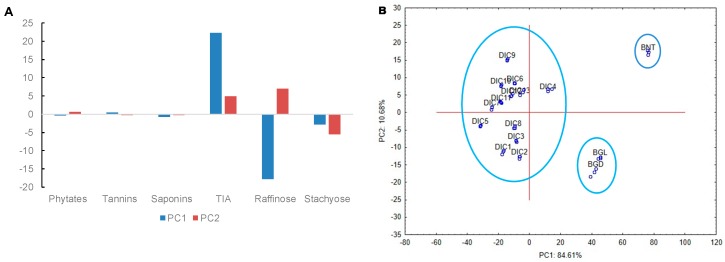
Principal component analysis. (**A**) Histogram of the variables contributing to PC1 and PC2, (**B**) Projection of the cases on the factor-plane. BNT—black bean without treatment, BGL—black bean germinated and lyophilized, BGD—Black bean germinated and dried at 50 °C.

Principal component analysis (PCA) is a statistical procedure that uses an orthogonal transformation to convert a set of observations of possibly correlated variables into a set of values of linearly uncorrelated variables called principal components. In this study, the first principal component (PC1) accounted for 84.61% of the total variability, while the second principal component (PC2) accounted for 10%. Figure 8A shows that trypsin inhibitory activity and raffinose concentration are the main responses contributing to the variability and construction of PC1. Similarly, those responses besides stachyose content accounted for the construction of PC2. PC analysis permitted to classify the obtained data into three groups. The first one contained observed responses in beans without treatment or BNT, meaning that this group has high values of trypsin inhibitors and low values of raffinose. In the second one, black bean sprouts dried either by convective air at 50 °C or lyophilized are grouped, indicating that there is no difference related to the drying method. Besides, BGL and BGD presented average values of TIA and raffinose, the responses related to PC1.

Finally, all DIC treated samples were grouped at the left side of PC1. TIA values related with this group were low, while raffinose ones were high. The separation in three well-differentiated groups indicated that all DIC treatments were different from germinated and also from non-treated beans.

## 3. Materials and Methods

Black bean seeds (*Phaseolus vulgaris* L.) were obtained in a local market of Mexico City. All reagents used were analytical grade from Sigma-Aldrich (St. Louis, MO, USA) and J.T. Baker (Phillipsburg, NJ, USA).

### 3.1. Germination

**Figure 9:**
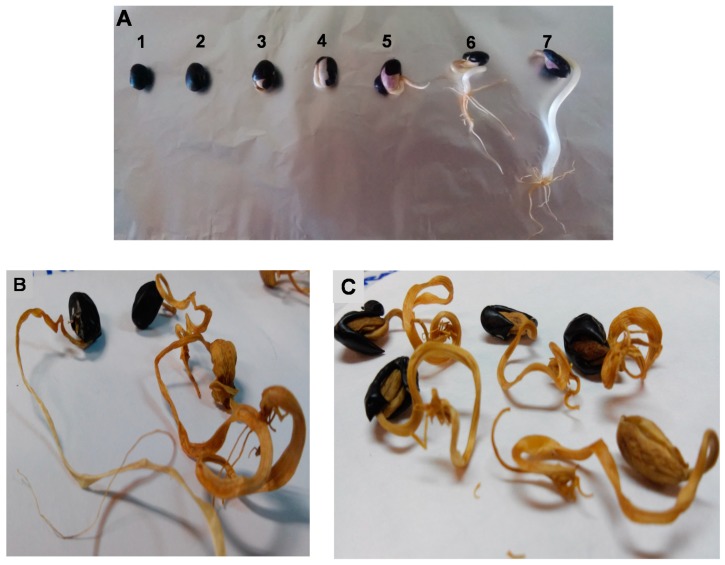
(**A**) germination of black beans (*Phaseolus vulgaris*) during seven days. (**B**) Sprouts of black bean dried at 50 °C (BGD) after 7 days of germination. (**C**) Sprouts of black beans lyophilized (BGL) after seven days of germination.

Germination process was realized as described by de Cortes Sánchez, et al. [46]. Seeds were selected according to their size, color, and absence of damage. Before germination, seeds were washed and disinfected with 10% NaOCl for 10 min and washed with water (four times, 10 min). Disinfected seeds were spread on trays covered with humid filter paper (Whatman, 50 × 50 cm), incubated at 23 ± 2 °C for 7 days (Figure 9A) into a germination chamber (Thermo Scientific 3110, Waltham, MA, USA) under environmental controlled conditions (22 °C and in darkness). Watering of the seeds during germination kept the paper always wet. Sprouts were separated into two batches; the first one was lyophilized (−48 °C/1.3 mBar. Labconco FreeZone 2.5, Kansas City, MO, USA) and named BGL (Figure 9B), the second batch was dried until humidity was 20% at 50 °C (Forced Air Drying Stove, Model 632 plus 65L–Tecnylab, Valencia, Spain); a sample was taken and named BGD (Figure 9C), the rest of the batch was used for DIC treatment. All samples were ground and kept in the absence of light until analysis.

### 3.2. DIC Treatment of Dried Black Bean Sprouts

**Figure 10:**
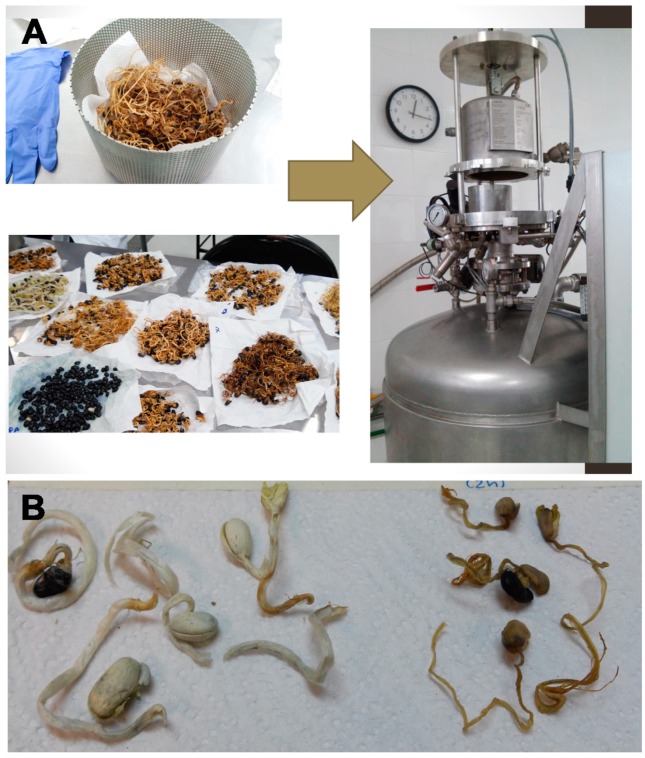
(**A**) Instant controlled pressure drop treatment. (**B**) DIC5, germinated beans treated under 0.27 MPa during 70 s.

An instant controlled pressure drop treatment (DIC) was carried out according to Guillamon, et al. [47], using a DIC-LABIC 0.1 DIC equipment (ABCAR-DIC Process, La Rochelle, France) following a central composite rotatable experimental design. Steam pressure (P) and thermal treatment time (t) were the independent variables. The design yielded 13 experiments with four (2^2^) factorial points, four star points (-α, -1, 0, +1, and +α) and five central points (0,0). Pressure values ranged from 0.10 to 0.30 MPa and treatment time from 10 to 80 s. The conditions of pressure and time used to treat black bean sprouts are described in Table 4. After DIC treatment, sprouts were dried at 50 °C until humidity was 9 ± 0.8%. Figure 10A shows the DIC equipment used to treat black bean sprouts, and Figure 10B illustrates DIC treatment 5 (DIC5) as an example of DIC treated samples.

### 3.3. Non-Nutritional Compounds Quantification

The concentration of saponins was determined according to the colorimetric method of Hiai, et al. [48]; results were expressed as mg diosgenin equivalent per g of sample. Phytates were evaluated by the method of Vaintraub and Lapteva [49] and reported as mg phytic acid equivalent per g of dry sample. The method of Folin–Ciocalteu [50] modified by Abdel-Aal and Hucl [51], was used to evaluate total phenolic compounds using a calibration curve of Gallic acid. The results were expressed as mg gallic acid equivalent per g of sample. Trypsin inhibitors were quantified by the method of Smith, et al. [52] with modifications, adding the enzyme in the last step, as suggested by Liu and Markakis [53]; results were reported as mg of trypsin inhibited per g of sample. Condensed tannins were quantified using (+)-catechin, and reporting results as mg catechin equivalent per 100 g of sample [54]. All absorbance readings were done in a MultiscanGo, (Thermo scientific, Waltham, MA, USA).

Oligosaccharide content was determined by HPLC using the method of Muzquiz, et al. [55] and [56] using an Agilent Technologies 1200 Series (Germany). Twenty-five µL of the sample were injected into a Zorbax Carbohydrate Analysis Column (150 × 4.6 mm, 5 μm, Agilent technologies, Germany). The mobile phase was acetonitrile: water (60:40 *v/v*) 0.8 mL/min. Raffinose, stachyose and verbascose were used as standards. Results were expressed as mg equivalents of the corresponding standard per g of sample.

### 3.4. Statistical Analysis

All determinations were done in triplicate. Results were the average ± standard deviation. ANOVA was applied to the results. Where differences were found, the Tukey HSD test was applied (*p* < 0.05) using Statistica Software (TIBCO® Data Science). After DIC treatment, non-nutritional compounds were compared against the pre-dried sample (BGD) using the Dunnet’s test. Finally, the Principal Components Classification was applied for all treatments.

## 4. Conclusions

The germination process increased the phenolics, tannins and saponins significantly in black bean seeds, while decreased phytate content and trypsin inhibitors. These changes were due to time and germination conditions. The combination of germination and controlled pressure drop reduced compounds that affect the quality of proteins such as tannins and increased those compounds that have beneficial effects on health such as oligosaccharides. DIC is an efficient alternative to make more available functional compounds in black bean sprouts.

## Figures and Tables

**Table 1:** Non-nutritional compounds in non-treated black beans (BNT), germinated and lyophilized (BGL), and germinated-dried at 50 °C (BGD) *.

Compounds	BNT	BGL	BGD
Phenolics ^1^	4.11 ± 0.07 ^a^	8.20 ± 0.32 ^b^	8.35 ± 0.38 ^b^
Phytic acid ^2^	17.33 ± 0.20 ^a^	12.80 ± 0.71 ^b^	13.44 ± 1.10 ^b^
Tannins ^3^	2.62 ± 0.06 ^a^	4.55 ± 0.20 ^b^	4.47 ± 0.25 ^b^
Saponins ^4^	7.05 ± 0.12 ^a^	9.37 ± 0.19 ^b^	9.47 ± 0.31 ^b^
TIA ^5^	99.65 ± 0.43 ^a^	58.11 ± 0.80 ^b^	53.07 ± 1.15 ^b^
Total oligosaccharides ^6^	44.43 ± 0.18 ^a^	48.50 ± 0.19 ^b^	47.01 ± 0.01 ^b^
Raffinose	6.26 ± 0.06 ^a^	28.95 ± 0.44 ^b^	27.37 ± 0.19 ^b^
Stachyose	33.11 ± 0.67 ^a^	19.55 ± 0.06 ^b^	19.64 ± 0.14 ^b^
Verbascose	5.06 ± 0.60	ND	ND

* Results are expressed as ^1^ mg gallic acid eq/g db, ^2^ mg phytic acid eq/g db; ^3^ (+) catechin eq/g db; ^4^ mg diosgenin eq/g db; ^5^ mg of pure trypsin inhibited (TIA)/g db; ^6^ Total oligosaccharides is the sum of the individual oligosaccharides measured and expressed in mg oligosaccharide/g db. Results are the average of three independent experiments ± DE. Different letters in a row represent statistical differences (Tukey HSD *p* < 0.05). ND—non detected.

**Table 2:** Non nutritional compounds concentration in germinated beans after controlled pressure-drop (DIC) treatment.

Treatment	Pressure	Time	Phenolics	Phytates	Tannins	Saponins	TIA
BGD	-	-	7.74 ± 0309	12.46 ± 0.20	4.15 ± 0.25	8.78 ± 0.39	53.07 ± 1.15
DIC1	0.30	45	8.20 ± 0.10 *	14.99 ± 0.13 *	1.14 ± 0.11 *	9.02 ± 0.10	15.30 ± 0.52 *
DIC2	0.20	45	6.95 ± 0.25 *	13.73 ± 0.32 *	2.05 ± 0.09 *	8.67 ± 0.09	25.59 ± 0.18 *
DIC3	0.20	45	6.98 ± 0.09 *	12.72 ± 0.16	1.96 ± 0.07 *	8.32 ± 0.04 *	24.40 ± 0.13 *
DIC4	0.10	45	6.63 ± 0.04 *	14.19 ± 0.17 *	3.10 ± 0.15 *	8.22 ± 0.07 *	49.62 ± 0.1.2
DIC5	0.27	70	8.29 ± 0.07 *	15.99 ± 0.22 *	2.11 ± 0.09 *	10.99 ± 013 *	2.20 ± 0.07 *
DIC6	0.27	20	6.98 ± 0.13 *	15.81 ± 0.15 *	1.79 ± 0.12 *	9.15 ± 0.07 *	28.72 ± 0.24 *
DIC7	0.20	80	8.27 ± 0.07 *	15.39 ± 0.51 *	2.72 ± 0.11 *	10.95 ± 0.06 *	10.63 ± 0.61 *
DIC8	0.20	10	6.67 ± 0.06 *	13.85 ± 0.11 *	2.21 ± 0.16 *	9.52 ± 0.12 *	23.18 ± 0.24 *
DIC9	0.20	45	6.81 ± 0.12 *	13.72 ± 0.17 *	2.11 ± 0.11 *	9.07 ± 0.05	21.72 ± 0.13 *
DIC10	0.20	45	6.70 ± 0.06 *	13.62 ± 0.15 *	2.11 ± 0.24 *	8.99 ± 0.05	21.11 ± 0.31 *
DIC11	0.20	45	7.25 ± 0.05 *	13.85 ± 0.13 *	2.00 ± 0.06 *	8.57 ± 0.28	19.16 ± 0.13 *
DIC12	0.13	20	6.46 ± 0.05 *	13.31 ± 0.24	2.97 ± 0.11 *	8.86 ± 0.16	25.58 ± 0.31 *
DIC13	0.13	70	6.59 ± 0.28 *	14.97 ± 0.17 *	2.29 ± 0.15 *	9.98 ± 0.09 *	31.38 ± 0.55 *

Pressure—Processing steam pressure in MPa. Time—Processing time in seconds. BGD—black bean germinated and dried at 50 °C. DIC1–13—black bean germinated and dried after DIC treatment. Phenolics concentration is expressed as mg Gallic Acid eq/g db. Phytates are expressed as Phytic acid eq/g db. Tannins are expressed as mg Rutin eq/g db. Saponins are expressed as mg Diosgenin eq/g db. TIA are expressed as mg of pure trypsin inhibited/g db. Results represent the average of three repetitions ± standard deviation. Means in a column marked with * are statistically different to control (BGD) by Dunnet, *p* < 0.05.

**Table 3:** Oligosaccharides concentration in black beans sprouts treated with controlled pressure drop (DIC).

Treatment	Pressure	Time	Raffinose	Stachyose	Total Oligosaccharides
BGD	-	-	27.37 ± 0.19	19.64 ± 014	47.01 ± 0.01
DIC1	0.30	45	71.61 ± 0.18 *	31.84 ± 0.81 *	103.45 ± 0.93 *
DIC2	0.20	45	66.04 ± 0.25 *	36.10 ± 0.28 *	102.14 ± 0.15 *
DIC3	0.20	45	68.70 ± 0.77 *	29.50 ± 0.39 *	98.20 ± 0.21 *
DIC4	0.10	45	69.53 ± 0.17 *	27.74 ± 0.13 *	97.27 ± 0.90 *
DIC5	0.27	70	82.21 ± 0.39 *	21.22 ± 0.43 *	103.43 ± 0.60 *
DIC6	0.27	20	80.32 ± 0.15 *	19.67 ± 0.45	99.99 ± 0.81 *
DIC7	0.20	80	81.51 ± 0.16 *	18.12 ± 0.56 *	99.63 ± 0.43 *
DIC8	0.20	10	72.23 ± 0.02 *	27.72 ± 0.09 *	99.95 ± 0.20 *
DIC9	0.20	45	81.80 ± 0.02 *	2.69 ± 0.33 *	84.49 ± 0.35 *
DIC10	0.20	45	84.80 ± 0.34 *	19.61 ± 037	104.41 ± 0.38 *
DIC11	0.20	45	81.89 ± 0.04 *	23.24 ± 0.07 *	105.13 ± 0.27 *
DIC12	0.13	20	79.12 ± 0.14 *	21.51 ± 0.26 *	100.63 ± 0.30 *
DIC13	0.13	70	75.68 ± 0.17 *	20.78 ± 0.04 *	96.46 ± 0.18 *

Pressure—Processing steam pressure in MPa. Time—Processing time in seconds. BGD—black bean germinated and dried at 50 °C. DIC1–13—black bean germinated and dried after DIC treatment. Raffinose is expressed as mg/g db. Stachyose is expressed as mg/g db. Total oligosaccharides is the sum of raffinose and stachyose. Results represent the average of three repetitions ± standard deviation. Means in a column marked with * are statistically different to control (BGD) by Dunnet, *p* < 0.05.

**Table 4:** Processing conditions for instant controlled pressure drop (DIC).

Treatment	Pressure (MPa)	Time (s)
DIC1	0.30	45
DIC2	0.20	45
DIC3	0.20	45
DIC4	0.10	45
DIC5	0.27	70
DIC6	0.27	20
DIC7	0.20	80
DIC8	0.20	10
DIC9	0.20	45
DIC10	0.20	45
DIC11	0.20	45
DIC12	0.13	20
DIC13	0.13	70
